# Book Reviews

**Published:** 1897-03

**Authors:** 


The Practice of Medicine. A Text-Book for practitioners and students, with special reference to diagnosis and treatment. By James Tyson, M. D., Professor of Clinical Medicine in the University of Pennsylvania, and Physician to the Hospital of the University; Physician to the Philadelphia Hospital; Fellow of the College of Physicians of Philadelphia, etc., etc. Octavo, pp. xvi.—1183. Illustrated. Philadelphia: P. Blakiston, Son & Company, 1012 Walnut street. 1896.

It has been thought by some, since the field of medicine has become subdivided into numerous specialties, that there would soon be no room for the so-called general practitioner; but the frequent appearance of works on the practice of medicine during the past few years would seem to indicate that such a belief is not well grounded.

The present treatise is announced as having been the result of several years' labor and as representing in a large measure personal work, though it does not pretend to be based entirely on personal practice.

Tyson opens his text-book with a section on infectious diseases, and proceeds at once to the consideration of typhoid fever. He places the bath treatment at the head of the remedies when it can be employed. Rest and liquid nourishment go hand in hand with it, but medicine plays but very little part in its management. He insists upon a careful watchfulness during the convalescence, and says that the specific treatment of the disease by serum is as yet tentative, and results admit of no definite conclusions. The advanced thought of the profession finds itself in close touch with Tyson on this subject.

Diphtheria is defined as an acute, contagious inflammatory disease due to inoculation with the Klebs-Lœffler bacillus, and especially characterised by the formation of false membranes and by secondary constitutional infection. We have given this in full because we regard it as about the best definition yet offered. Tyson holds to three principal indications in the management of diphtheria—namely: first, to prevent constitutional infection; second, to support the system; and third, to combat the toxins. This picture should be kept in the mind of every physician called upon to minister to this malady.

The second section considers diseases of the digestive system, and diseases of the stomach occupy necessarily the largest space in this subdivision. In late years probably more attention has been bestowed on gastric disorders than upon those of any other portion of the digestive tract, because it is in the stomach that many wasting diseases originate. To correct the early faults of digestion should be the first duty of the physician, lest neglect here may lead to future severe, if not irremediable, complications. Tyson has dealt with the subject very ably and with due conservatism. In the subdivision devoted to the consideration of appendicitis will be found all that can be said under its medical management. This being essentially a surgical disease, Tyson here very properly directs that as soon as the diagnosis of appendicitis is established, indeed pending its settlement, a competent surgeon should be associated with the physician, for the reason that in the vast majority of cases operative treatment is sooner or later demanded, while the necessity for such treatment is best settled by daily conference. This is most sensible advice, and when the profession can be made to accept it as a governing principle appendicitis will be less destructive. Intestinal obstruction is always an important subject and one that requires great skill in diagnosis. Tyson has written many practical sentences on this branch of the subject and utters the dicta of experience.

The third section takes up diseases of the respiratory system. Pneumonia always claims an important place under this head, and Tyson has not neglected to give it consideration in accordance with its importance. He advocates general blood-letting in proper cases, and dry cupping when adynamia is too great to admit of the former. Bleeding he believes not only relieves distressing symptoms, but lessens the crisis and shortens the disease. We are of the opinion that this remedy should be employed with great discretion and limited to robust young subjects who become victims of this disease.

Pulmonary tuberculosis Tyson hesitates not to state is an infectious disease due to the lodgment and proliferation of the tubercle bacillus in the lung substance. He believes it to be an infectious disease, the route of invasion being either by the air-passages or the blood. This is a sound position on which to anchor, and will lead the profession out of a darkness that has heretofore shrouded the malady. We do not mean to be understood that this attitude of Tyson is new, but rather that it is becoming more and more accepted by experienced clinicians, of which Tyson may be accepted as an exemplar.

The fourth section takes up for consideration diseases of the heart and blood-vessels. A study of these affections always possesses extreme interest, and any new light is more than welcome. The neuroses of the heart are briefly but ably handled in this treatise, and Tyson displays in their consideration not only a knowledge of the subject but discretion in handling it. The sensible, though brief, consideration of the treatment of tachycardia, brachycardia, stenocardia and arythmia is commended to the searcher for information on these subjects. Arterial sclerosis is one of the most important diseases of the blood-vessels, and is very ably and fully discoursed upon.

In section five, diseases of the blood and blood-making organs are taken up and dealt with understandingly. Progressive pernicious anemia is one of the most subtle and difficult maladies with which a physician is called upon to cope, and what Tyson has to offer on the subject is clear and helpful, if not an entirely satisfactory solution of the problem. As a drug, arsenic has always been a chief reliance, and Tyson so regards it ; but it must be supported by other hygienic and food aids in order to reap substantial benefit from its employment.

A short section, number six, is devoted to the consideration of diseases of the thyroid gland, in which myxedema and cretinism are considered under separate subdivisions.

Then comes section seven, in which diseases of the urinary organs are taken up, and here is afforded an opportunity for a clinician like this author to display his skill and knowledge. As a test for albumin, Tyson regards the heat and nitric acid tests as the most delicate and reliable. It should, however, be used under skilful hands in order to yield the most reliable information. Diseases of the kidney are dealt with in considerable detail, and while diseases of the bladder occupy less space they are yet presented with much clearness and satisfaction.

In section nine we find constitutional diseases, such as rheumatism, gout, diabetes mellitus, incipient lithemia, and the like, dealt with in considerable detail as well as with skilful judgment.

Section ten, in which diseases of the nervous system are considered, is, as might be expected, a long one, since the nerves themselves permeate every fiber and organ ; and this, we presume, is the reason why nerve specialists, at least some of them, have appropriated nearly every part of the body to themselves in the practice of their chosen field of specialism. If the general practitioner does not look out betimes he will find himself supplanted by the specialist in nervous diseases. However, Tyson has presented this part of his treatise in minute detail and in a fashion that will commend itself both to the general physician and nerve specialist.

The closing sections present many of those diseases not included in the foregoing general heads. For instance, section eleven treats of diseases of the muscular system ; section twelve, of the intoxications ; section thirteen, of the effects of exposure to high though bearable temperature ; section fourteen, of animal parasites and the conditions caused by them ; section fifteen, contains a summary of symptoms following over-doses of poison, alphabetically arranged. To this is added a table of minimum dose which has caused death, and maximum dose followed by recovery. In an appendix is given a table for reducing the metric system into English, and one to convert degrees of the Fahrenheit thermometer to centigrade, and vice versa. A well-prepared index closes the volume.

It is quite in keeping with the proprieties of the occasion, and it is without detraction from the merit of any other recent treatise on medicine, that we venture the opinion that Tyson's work will not only prove a text-book in name, but will become one in reality. The general arrangement of the treatise is such as appeals to the modern-trained practitioner of medicine, and the clinical facts recorded in it possess a clearness and value that will promptly bring it to the front of medical literature. The publishers have not neglected to perform well their part in the making of a handsome and useful treatise on medicine.

THE AMERICAN YEAR-BOOK OF MEDICINE AND SURGERY. Being a Yearly Digest of Scientific Progress and Authoritative Opinion in all Branches of Medicine and Surgery, drawn from Journals, Monographs and Text-Books of the leading American and Foreign Authors and Investigators. Collected and arranged with Critical Editorial Comments by J. M. Baldy, C. H. Burnett, Archibald Church, Arthur H. Cleveland, Colman W. Cutler, J. Chalmers DaCosta, W. A. N. Dorland, Louis A. Duhring, V. P. Gibney, Homer W. Gibney, Henry A. Griffin, John Guitéras, C. A. Hamann, H. F. Hansell, B. C. Hirst, E. Fletcher Ingals, W. W. Keen, H. Leffman, Henry G. Ohls, H. T. Patrick, William Pepper, Wendell Reber, D. Riesman, Louis Starr, Alfred Stengel, G. N. Stewart, and Thompson S. Westcott. Under the general editorial charge of GEORGE M. GOULD, M. D. Profusely illustrated. Royal 8vo, pp. 1257. Philadelphia: W. B. Saunders. 1897.

This is the first of the year-books to present itself, and it would seem not only on this account, but for the further reason that it is so comprehensive in plan and so elaborate in detail, that it also presents a first claim upon time and space.

The present volume is constructed upon the precise lines of the first one issued, which appeared last year. Some of the editors have been changed, but out of the total number, twenty-eight, seventeen are Philadelphians. We suggested last year that it would seem wise for the editor-in-chief to distribute his associates over a wider section of the country, with the object of giving his book a greater national identity. We still think this suggestion a wise one.

The immensity of this work is something almost startling, and betrays an indefatigable industry on the part of the editor-in-chief and his associates that is worthy of praise and imitation. The accuracy of the references made at the foot of each page has been tested in a number of instances at random and we have not been able to detect an error. It is a fact that should be remembered regarding works of this kind that they interfere in no sense with the subscriber's necessity for medical journals. This book rather accentuates the importance of taking a liberal number of medical journals, for when one sees in it a reference relating to a subject in which he is interested he regrets that he is not able to lay his hand upon the original journal quoted in his own library. We mean to be understood as saying that this work and those of a similar nature accentuate the necessity of possessing the leading medical journals. It is a satisfaction to the Buffalo Medical Journal to find itself often quoted in this work, and we trust that its columns will prove of sufficient interest to warrant a continuation of frequent reference to them. A busy physician needs to find a subject quickly, and this year-book is one of the best indexes to the literature of 1896 that a physician could possibly possess. The summary at the head of each article giving the direction and results of the year's work is an interesting feature which will undoubtedly prove useful.

We could wish that the editor had not seen fit to drag into his preface reference to a controversy that the profession in general has very little interest in. There are always two sides to every question, and we have no doubt the publishers referred to considered their course justifiable.

The index of this work is remarkable for its comprehensiveness. There are sixty triple-column pages of it that enables one to make quick reference to any subject between its covers.

The book is well printed, but it is rather heavy for cloth binding. We advise purchasers to procure it in leather. We shall look forward each year to the advent of this admirable compilation of the work for the preceding twelve months.

---

Report of the Commissioner of Education for the Year 1894-95. In two volumes. Washington: Government Printing Office. 1896.

This report has been received with greater promptitude than usual, which is a commendable fact. We have heretofore pointed out in these columns that the value of these reports was detracted from to a considerable degree by their tardiness, and we are pleased to note a disposition to improve upon the old slow-going fashion in regard to this matter.

In the chapter on professional education, by Dr. A. Erskine Miller, is to be found a grouping of interesting data regarding the requirements for the practice of medicine in the several states. The author of this chapter seems to be pleased because there are more medical students, in proportion to population, in this country than in any other part of the civilised world. He says:

> The people themselves are making no complaints that there are too many physicians; they content themselves with the thought that it is the same with physicians as with any commodity—the greater the supply the less the cost. The medical men themselves are the only ones who have any reason to complain, and is it not probable that some of them are falling into the way of some trades unions in endeavoring to shut out accessions, in order that they may enjoy exclusive possession —and monopoly?

We are surprised that any professional man, and especially a physician of sufficient education to justify his appointment to an important place under the department of education, should be allowed to print such rot in a document issued and sanctioned by the government, at a time, too, when every country in the civilised world is doing its utmost to establish and maintain a high standard in the profession of medicine. It smatters too much of quackery to find such doctrines put forth by a government official.

The general work of the commissioner of education is to be commended, and in the volume before us appears a full record of the work of his department for the year to which it pertains.

---

TRANSACTIONS OF THE TEXAS STATE MEDICAL ASSOCIATION. Held at Fort Worth, Texas, April 28, 29, 30 and May 1, 1895. Printed by order of the association. 1896.

The Texas State Medical Association has always been famous for the excellent character of its work as told in its annual volume of transactions, which is a welcome visitor to the library of every one that takes an interest in medical progress. It is an interesting fact that the several state medical societies are now contributing more to the general *esprit de corps* of the profession than any other organisations, and nothing bespeaks the progressive spirit of a physician in a higher degree than a manifestation of an active interest in his state medical society. He should not only attend its meetings with regularity, but he should secure its volumes of transactions and keep them within easy reach for reference on his library shelves.

The volume under consideration contains many interesting scientific papers and discussions upon the same. A list of members of the association, of auxiliary societies in affiliation with the state society, and of the officers of the association since its formation is given. This volume is worthy of a more permanent cover. Paper furnishes very poor binding for a medical book that is needed for frequent reference.

---

ANATOMICAL ATLAS OF OBSTETRIC DIAGNOSIS AND TREATMENT. By OSCAR SCHAEFFER, M. D. Small octavo, pp. xii.—234. Illustrated. New York: William Wood & Co. 1896.

This volume, to borrow the author's own words, deals especially with the morphology of the female pelvic organs as the anatomical basis in the physiological and pathological phenomena of pregnancy and labor.

Schaeffer has presented the matter in a most interesting fashion and has displayed artistic talent and anatomical accuracy in his engravings. The drawings are quite original, and are many of them printed in colors. It is a book that may be regarded as a valuable aid to the study of pregnancy and labor which every teacher will appreciate. The work closes with a section on general observations on obstetric therapeutics, in which the most useful obstetrical remedies with their dosage and therapeutic indications are given, as well as directions for obstetric antisepsis. It is a work that should find its way into the library of every practical obstetrician.

---

A TEXT-BOOK FOR TRAINING SCHOOLS FOR NURSES, including Physiology and Hygiene and the Principles and Practice of Nursing. By P. M. WISE, M. D., Medical Superintendent St. Lawrence Hospital; Editor of *The State Hospitals Bulletin*, etc. With an introduction by Edward Cowles, M. D., Physician-in-Chief and Superintendent of the McLean Hospital, Boston, Mass. In two volumes. New York: G. P. Putnam's Sons. 1896.

Many text-books on the subject of nursing have heretofore been published, but none has attempted to cover the field in the same manner as does the one before us. In addition to the preliminary work prescribed, and the routine care detailed during an ordinary course of training, we find in this work explicit directions with reference to the special care of the insane. The author, Dr. Wise, is an expert alienist, and is now at the head of the lunacy commission in this state. He is, therefore, a competent authority to write on this latter branch of the subject. But he is, moreover, well qualified to teach nurses in all the departments in which they may be called upon to serve, for he was a well-trained physician before he became an alienist; and here we may be permitted to remark that this is the only proper way to become skilful in the management of the insane. A man must first be a competent physician and acquire that competency by several years' practice before taking up insanity as a specialty.

An appendix to the work gives, first, a table of weights, measures and symbols; a table of drugs, doses, action and uses; alimentary preparations for the sick; and finally a glossary of technical words and terms, the whole followed by a very complete general index.

We believe this treatise will be adopted as a text-book in every training-school in this country. Its excellence is such as to justify this belief, and surely training-schools cannot afford to dispense with the best.

---

ANNUAL REPORT OF THE STATE BOARD OF CHARITIES FOR THE YEAR 1895. Transmitted to the legislature, March 25, 1896. Albany, N. Y.: Wynkoop Hallenbeck Crawford Company, State Printers. 1896.

The State Board of Charities always issues an interesting report. The Soldiers and Sailors' Home at Bath is the first institution considered in this volume, and the president of the board, William R. Stewart, Esq., gives the results of his inspection December 5, 1895. At that time there were 1,267 inmates in the institution, including fifty-seven officers and employees.

The hospital contains eight wards, furnishing 180 beds, 130 of which were occupied. It is stated that from eighty to 100 deaths a year occur at the home.

In connection with this institution we are pleased to note that Col. Chas. O. Shepard, of Buffalo, has lately been appointed its superintendent, and we feel sure that under his administration the home will take rank second to no charity of its class in this country.

Dr. Chas. S. Hoyt, superintendent of state and alien poor, remarks in his report that the number of alien paupers removed from the poor-houses, alms-houses and other charitable institutions and sent to their homes in the different countries of Europe during the fiscal year ending September, 1895, was 261, *i. e.*, to England, 41 ; to Ireland, 37 ; to Germany, 53 ; to Austro-Hungary, 31 ; to Italy, 88 ; to Russia, 7 ; and to Holland, 41. Their condition when landing in this country was as follows : Crippled, 17 ; paralytic, 9; epileptic, 7 ; aged, infirm and decrepit, 22 ; feeble-minded, 63 ; vagrant and variously diseased, 98 ; otherwise disabled, 48.

The per capita expenditure necessary to the deportment of these people was $20.45. This is an economical way to dispose of a class of individuals that is being unloaded upon this country through the mistaken kindness of friends or the inadequate circumspection of immigration officers.

An elaborate report is made by the Hon. Wm. P. Letchworth, concerning the poor-houses in the 8th judicial district. Commissioner Letchworth also presents a brief report concerning the Thomas Asylum for orphans and destitute Indian children.

In many other respects this report is one of interest.

---

Eleventh Annual Report of the State Board of Health and Vital Statistics of the Commonwealth of Pennsylvania. Transmitted to the Governor December 1, 1895. Pennsylvania: Clarence M. Busch, State Printer. 1896.

The report of this health board is not unlike those of other states in most respects. It, however, not only contains its own by-laws, but a grouping of the laws of the commonwealth of Pennsylvania relating to the protection of life and health passed at the legislative session of 1895. From it we learn that Pennsylvania has established a state board of veterinary medical examiners, whose provision it is to regulate the practice of veterinary medicine and surgery in the Keystone state. In this respect it seems to have followed pretty closely the example of New York. A number of other laws relate to the prevention and control of infectious diseases ; the regulation and sale of poisons ; the prevention of pollution of water streams ; the regulation of house-drainage and sanitation ; the promotion of cleanliness in public schools ; to provide against the adulteration of food, and many other important laws looking toward the prevention of disease.

While it is an admitted fact that in public sanitation Pennsylvania has not kept pace with many other states, yet now she is evincing a spirit of progress in this respect that is commendable. This report indicates an activity all along the lines of public health protection and the prevention of disease, and is a credit to the officers who are mainly responsible for the volume, especially the secretary, under whose direction it has been prepared.

---

Essentials of Physical Diagnosis of the Thorax. By Arthur M. Corwin, A. M., M. D., Demonstrator of Physical Diagnosis in Rush Medical College, etc. Second edition, revised and enlarged. Philadelphia: W. B. Saunders, 925 Walnut street. 1896.

The author of this useful aid to the study of diagnosis of diseases of the thorax has availed himself of the opportunity afforded by a second edition to revise the original text and to add a section setting forth the signs found in diseases of the chest. It is really an outline of the essentials of the science of physical diagnosis as applied to the thorax. These essentials have been grouped in an appropriate manner conforming to the topography of the several regions, and the whole arrangement is such as to aid the student of internal medicine. We regard it as a useful guide to the study of diseases of the thorax.

Transactions of the Medical Society of Virginia. Twenty-sixth Annual Session, held at Wytheville, Va., September 3, 4 and 5, 1895. Landon B. Edwards, M. D., Secretary. Richmond: J. W. Ferguson & Son, Printers. 1895.

The Medical Society of the Old Dominion has long been distinguished for the high character of its scientific work. The volume before us contains many valuable paper, among which may be mentioned the president's address on the Intellectual life of the medical man; Dr. John Herbert Claiborne's paper on The Old Virginia Doctor; Diagnosis and treatment of cholelithiasis by Dr. George Ben Johnston, of Richmond. A biographical register of the fellows of the society forms an interesting supplement to the volume. The secretary, Dr. Landon B. Edwards, of Richmond, publishes a prefatory note, in which he accounts for the delay in the issue of the volume through the fact that authors did not forward their manuscripts promptly, and he adds that authors who expect their papers to receive attention should not take their manuscripts away from the desk of the secretary. He also says that some of the proof-sheets sent by courtesy to the authors were never returned. This admonition would have a forceful application in the ranks of some other medical associations.

---

Transactions of the American Gynecological Society. Volume XXI. For the Year 1896. Philadelphia: Wm. J. Dornan, Printer. 1896.

The volume before us is a tangible evidence of the continued prosperity of this society, which is yearly working along the lines of advancement in its chosen field of research. One of the most interesting papers in this book is by J. Clifton Edgar on Aids in obstetric teaching. This is a department in which the author of the paper is distinguishing himself and in which he bids fair to become a leader. There is a constantly-increasing need for improvement in medical teaching, and especially in the obstetric field. Physicians of experience and observation are gradually reaching the conclusion that no one ought to be permitted to begin the practice of medicine who has not had an under-graduate experience of at least thirty cases of labor.

It was fitting that at this meeting the distinguished founder of the association, Dr. James R. Chadwick, of Boston, should be chosen president in commemoration of the twentieth anniversary of the society. The volume closes with memorials of Robert Battey and Thomas Kieth.

---

Transactions of the Medical and Chirurgical Faculty of the State of Maryland. Ninety-eighth Annual Session, held at Baltimore, Md., April, 1896, also Semi-Annual Session, held at Belair, Md., November, 1895, and Special Memorial Meeting in Baltimore, December 14, 1895. Baltimore, Md.: The Deutsch Lithographing Company.

This book is issued by one of the oldest state medical organisations in the country, yet it is about the smallest volume that any of them sends forth. It contains minutes of the proceedings, the president's annual address and the annual oration, together with reports of committees and lists of officers and members, to which is added in this volume an address by Dr. Chadwick, of Boston, on medical libraries, their development and use, delivered at the formal opening of the new hall and library, January 11, 1896. Th scientific papers read and discussed at the meeting, for some inexplicable reason, are not published.

TRANSACTIONS OF THE COLORADO STATE MEDICAL SOCIETY. Twenty-sixth Annual Convention. By-Laws and List of Members. Denver, June, 1896. Published for the Society. Denver, Col.: The Smith-Brooks Printing Company. 1896.

Colorado seems to possess an uncommonly active and progressive state medical society. We have heretofore had occasion in these columns to refer to the value of the work done at its meetings and the excellent form in which the proceedings are published. The present volume indicates that no step backward has been taken by this society. The book is full of interesting, well-written papers, and some of the discussions are extremely spirited and creditable. We bespeak continued success for the society, that now numbers about 300 members.

